# Nuclear medicine in the operating room: present and future

**DOI:** 10.1093/bjr/tqag073

**Published:** 2026-03-31

**Authors:** Renato A Valdés Olmos, Angela Collarino, Daphne D D Rietbergen, Giusi Pisano, Lenka Pereira Arias-Bouda, Francesco Giammarile, Sergi Vidal-Sicart

**Affiliations:** Interventional Molecular Imaging Laboratory, Leiden University Medical Centre, Leiden 2333 ZA, The Netherlands; Department of Radiology, Section of Nuclear Medicine, Leiden University Medical Centre, Leiden 2333 ZA, The Netherlands; Nuclear Medicine Unit, Department of Diagnostic Imaging and Oncological Radiotherapy, Fondazione Policlinico Universitario A. Gemelli IRCCS, Rome 00168, Italy; Interventional Molecular Imaging Laboratory, Leiden University Medical Centre, Leiden 2333 ZA, The Netherlands; Department of Radiology, Section of Nuclear Medicine, Leiden University Medical Centre, Leiden 2333 ZA, The Netherlands; Interventional Molecular Imaging Laboratory, Leiden University Medical Centre, Leiden 2333 ZA, The Netherlands; Nuclear Medicine Unit, Department of Diagnostic Imaging and Oncological Radiotherapy, Fondazione Policlinico Universitario A. Gemelli IRCCS, Rome 00168, Italy; Department of Radiology, Section of Nuclear Medicine, Leiden University Medical Centre, Leiden 2333 ZA, The Netherlands; Nuclear Medicine and Diagnostic Imaging Section, Division of Human Health, Department of Nuclear Sciences and Applications, International Atomic Energy Agency (IAEA), Vienna 1400, Austria; Department of Nuclear Medicine, Hospital Clinic Barcelona, Barcelona 08036, Spain; Institut d’Investigacions Biomèdiques August Pi i Sunyer (IDIBAPS), Barcelona 08036, Spain

**Keywords:** interventional nuclear medicine, SPECT/CT, PET/CT, radioguided surgery, sentinel node procedure, detection devices in operating room

## Abstract

The current role of nuclear medicine in the operating room is closely related to the enhancement of the sentinel node (SN) procedure developed more than 3 decades ago. At that moment, the so-called triple approach (lymphoscintigraphy, gamma probe detection, and blue dye) was established as the standard of care for SN biopsy in melanoma and breast cancer. It also marked a first international multidisciplinary learning effort based on skill transfer and outcome evaluation, laying the fundaments to delineate the emerging field of interventional nuclear medicine (iNM) thanks to a similar multimodality approach and multidisciplinary practice. Currently, imaging modalities like SPECT/CT and PET/CT allow to preoperatively generate precision roadmaps for navigation in the operating room. The combination of modern technologies has facilitated iNM incorporation for surgery in more complex anatomical areas. The SN procedure has been expanded to different gynecological and urological malignancies as well as head and neck and gastrointestinal cancers. Besides iNM for open surgery, it is also possible to guide robot-assisted laparoscopic procedures for both SN biopsy and resection of oligometastases. The increasing development of hybrid tracers for combined radioguidance and fluorescence will reinforce the role of iNM in the future. The aim of this review is to comprehensively evaluate the contribution of nuclear medicine concerning current clinical applications and technological advances in the field of radioguided surgery.

## Introduction

Sentinel node (SN) surgery was not the first operating room procedure to involve interventional nuclear medicine (iNM). In the past, radioguided surgery (RGS) played an important role in tumor resection.^1^ However, the introduction of the SN procedure highlighted the value of combining nuclear medicine imaging with RGS and marked a major step in cancer surgery. Moreover, lymphoscintigraphy was probably the key to the evolution of the SN from static to dynamic concept. In the era of elective lymph node dissection, Holmes et al^2^ started with the preoperative use of lymphoscintigraphy to make this approach more personalized for the surgical management of melanoma. By injecting the radiotracer at the primary tumor, it became evident that lymphatic drainage did not involve the entire nodal basin but rather a single or a small number of lymph nodes. On a functional basis, the SN concept identifies the lymph nodes that receive direct lymphatic drainage from the injection site as SNs, making them the most likely to harbor the earliest metastases.^3^ Nowadays, this dynamic SN concept constitutes the fundament for the surgical procedure in several solid malignancies. In all these applications, iNM plays an essential role, combining preoperative imaging with intraoperative detection following one to a few local radiopharmaceutical injections depending on the clinical indication.^4^ This role was reinforced with the development of equipment for preoperative imaging such as single-photon emission computer tomography (SPECT)/CT and positron emission tomography (PET)/CT.^5^ Whereas for PET/CT a separate scanner needs to be utilized, SPECT/CT is today generated with the same gamma camera used for lymphoscintigraphy. In this context, for preoperative nuclear medicine imaging lymphoscintigraphy, essential to assess drainage patterns, is considered as the first phase of the gamma camera procedure, and SPECT/CT as the second phase, important to anatomically localize SNs and detect other ones. Concerning intraoperative detection, the introduction of portable gamma cameras (PGC) and other tools, in addition to handheld gamma probes, has contributed to improve iNM utilization for RGS.^6^ In precision cancer surgery, radiopharmaceuticals play a pivotal use. Since many years, these radiopharmaceuticals, in the form of radiocolloids, have enabled to reproduce lymphatic drainage after local administration around the primary tumor and therefore are currently applied for SN surgery. In recent years, the addition of fluorophores like indocyanine green (ICG) to radiopharmaceuticals has resulted in hybrid tracers. These have been useful for SN search in areas of complex anatomy using bimodal equipment with near-infrared (NIR) cameras supporting detection together with gamma devices.^6^ Besides locally administered radiocolloids for SN surgery, there are radiopharmaceuticals for systemic administration which show preferential accumulation in target tumor lesions. Thus, facilitating their visualization by SPECT/CT or PET/CT and their subsequent resection by RGS in the operating theater.

**Figure 1:**
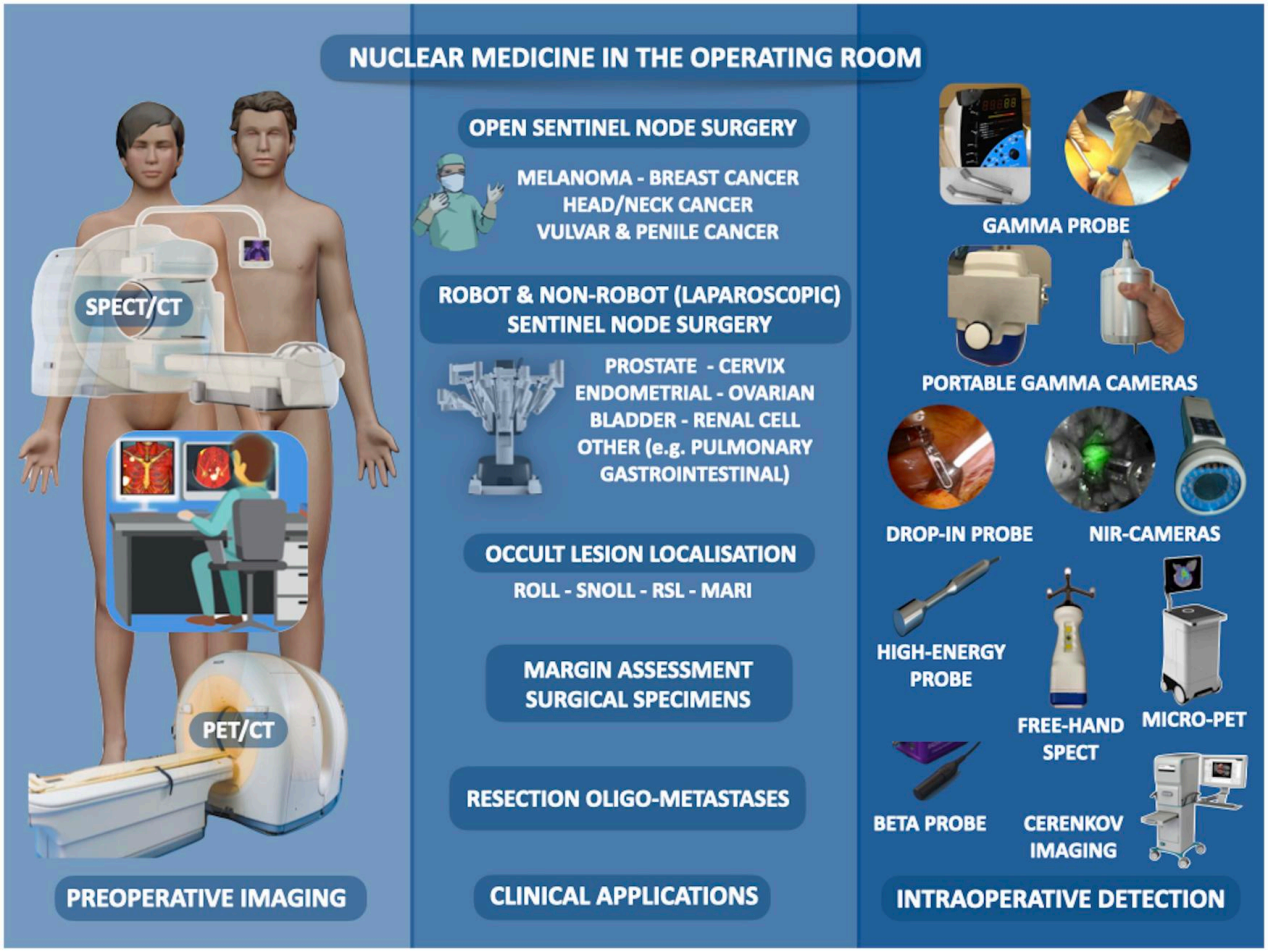
Nuclear medicine in the operating room. In the middle, an overview of the current clinical applications for which preoperative imaging roadmaps are generated by SPECT/CT or PET/CT (on the left) for further radioguided intervention in the operating room. On the right most available portable devices for intraoperative radioguided detection.

**Figure 2:**
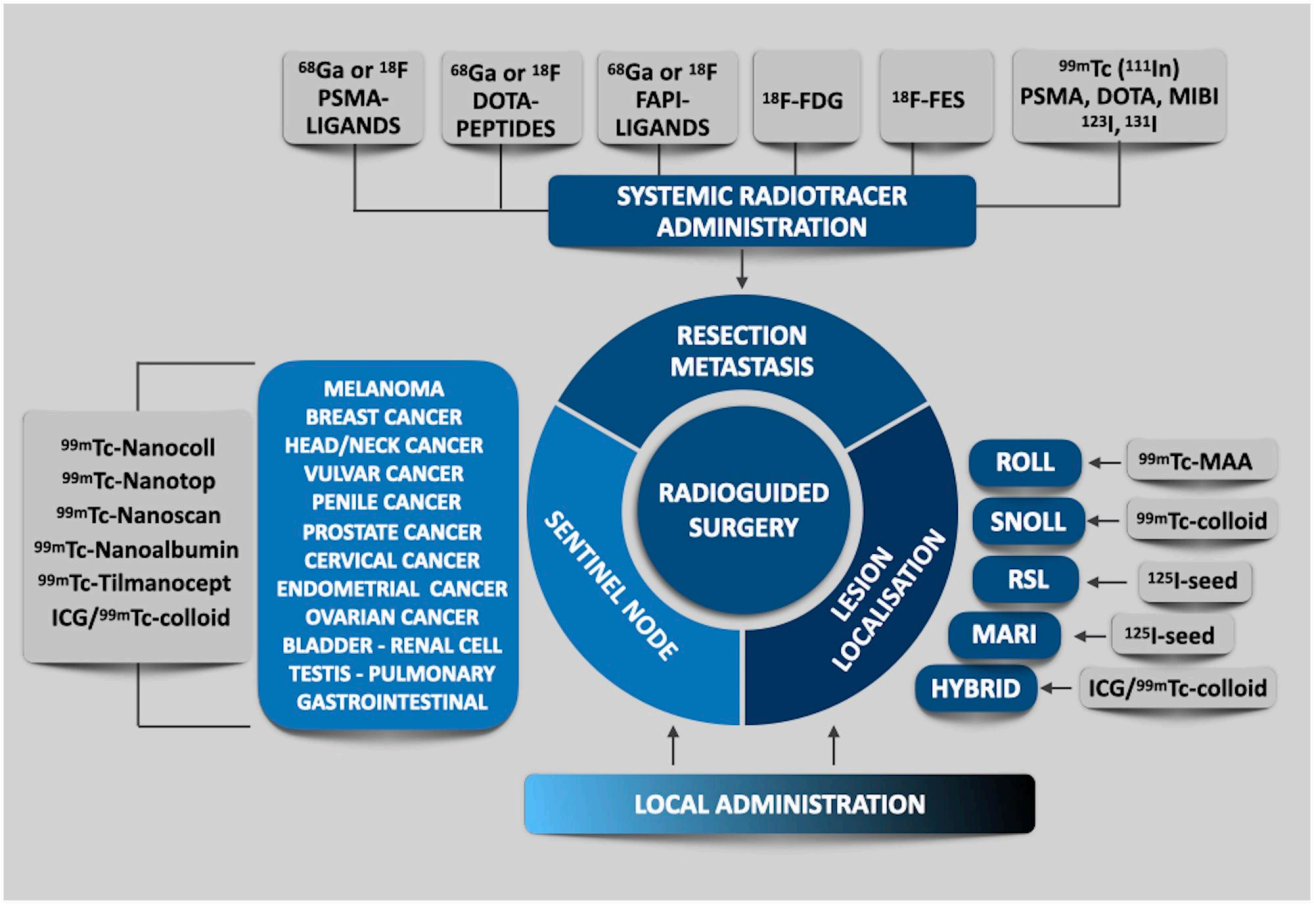
Schematic overview showing most frequent radioguided possibilities today. In the 2 lower sections the applications for sentinel node surgery and intraoperative lesion localization are displayed in relation to the local administered radiopharmaceuticals or implanted radioactive seeds. The upper section is related to the systemic administration of PET or SPECT tracers for radioguided resection of (oligo)metastases.

For precision cancer surgery, SPECT/CT and PET/CT enabled to preoperatively generate precision navigation roadmaps which are crucial for target detection during the surgical act. The combination of these tools with intraoperative technologies has facilitated iNM incorporation for SN surgery in different gynecological, urological, head and neck and gastrointestinal cancers (Figure 1). Besides iNM for open surgery, it is possible to guide robot-assisted laparoscopic procedures for both SN biopsy and radio-tagged resection of oligometastases as well as for primary tumor resection in the frame of specimen margin assessment tool.^7^ Based on these increasing clinical applications, both international nuclear medicine and oncological associations have generated guidelines and recommendations to implement training and homogenize practice for SN surgery and iNM.^8^ It is important to emphasize that learning and practicing each of these applications require a multidisciplinary approach and follow a multimodular training model. Figure 2 shows the different fields of iNM based current applications for minimally invasive cancer surgery. The principal objective of the presented work is to comprehensively review the contribution of iNM and RGS in the light of the current clinical application and the technological advances in this field.

## Interventional nuclear medicine in sentinel node surgery

Most clinical indications for SN biopsy were discussed and agreed on by several oncologic and nuclear medicine associations as well as in consensus expert meetings. To understand the possibilities of iNM for the SN procedure it is necessary to learn the mechanisms and routes of metastasis through the lymph-vascular system. The current TNM classification includes not only SN findings, but also the routes of lymphatic drainage from the site of primary tumors and further dissemination.^9^ In general, agreement was reached on most clinical indications for SN biopsy concerning early cancer stages without evidence of regional lymph node metastases (N0). In this section, the role of iNM and RGS for these applications will be reviewed in the light of multidisciplinary recommendations.^10^

### Most common applications: melanoma and breast cancer

The 2 most common applications of iNM-guided SN surgery are cN0 melanoma (T1b, T2, or T3; Breslow thickness of 0.8-4 mm) and cN0 breast cancer (T1 or T2). For these applications nuclear medicine imaging with a gamma camera and RGS using a gamma probe became integrated elements following the introduction of the so-called triple approach (lymphoscintigraphy, gamma probe detection, and blue dye) in the procedure for SN biopsy early in the 1990s. This resulted in a multimodality approach which was extensively evaluated during the period 1992-2012, showing high efficacy (pooled SN identification rates of 95% for breast cancer and of 98% for melanoma) and reliability (pooled false negative rate of 1.5% for breast cancer and of 2.6% for melanoma) in 154 studies (88 breast cancer, 66 melanoma) including more than 44 000 patients. Since then, it has been considered as the methodological standard of care in the clinical routine for SN biopsy.^11^

For melanoma, depending on its skin localization, the typical lymphatic drainage routes following intradermal radiocolloid injections around the primary lesion or around the excisional site may concern the groins, axillae, and neck. Less common routes are the epitrochlear/epicondylar for melanomas of hands/forearms and the popliteal for those of foot/leg. For melanomas of the trunk besides groin or axilla, the drainage to lymph nodes of the triangular intermuscular space on the back, bicipital sulcus and subcutaneously in the flank or adjacent to the areola may occur. For head melanomas, frequent routes are the periauricular, parotid, submandibular and cervical regions.^12^

For breast cancer, the principal lymphatic route is the axilla and approximately 90% of the SNs are found in level I which concerns the nodal external mammary, lateral axillary vein, subcapsular and axillary vein groups. Outside the axilla, the most important lymphatic route is the internal mammary chain with drainage also to the intramammary, interpectoral, and supraclavicular lymph nodes. Visualization of lymphatic drainage depends on the injection technique. When the radiocolloid is superficially administered related to the areola or the skin, drainage is almost invariably seen only to the axilla; whereas, using deep tumor related tracer injection, both axillary and non-axillary drainage are observed.^13^

Preoperative lymphatic mapping using lymphoscintigraphy has proven essential in melanoma and breast cancer for both the identification of the number of lymph nodes on the direct drainage pathway and the anatomical localization of those SNs. The preoperative imaging is strongly recommended due to the individual variability in lymphatic drainage. Lymphoscintigraphy results in a more effective handling of the gamma probe in the operating room and their combination enables to identify SNs in almost all patients.^10^ This combination improves not only the accuracy of the procedure, but does also reduce its morbidity when compared with the use of the gamma probe alone.^11^ Preoperative imaging also serves as quality control and management on the possible failure of the radiopharmaceutical injection.

**Figure 3:**
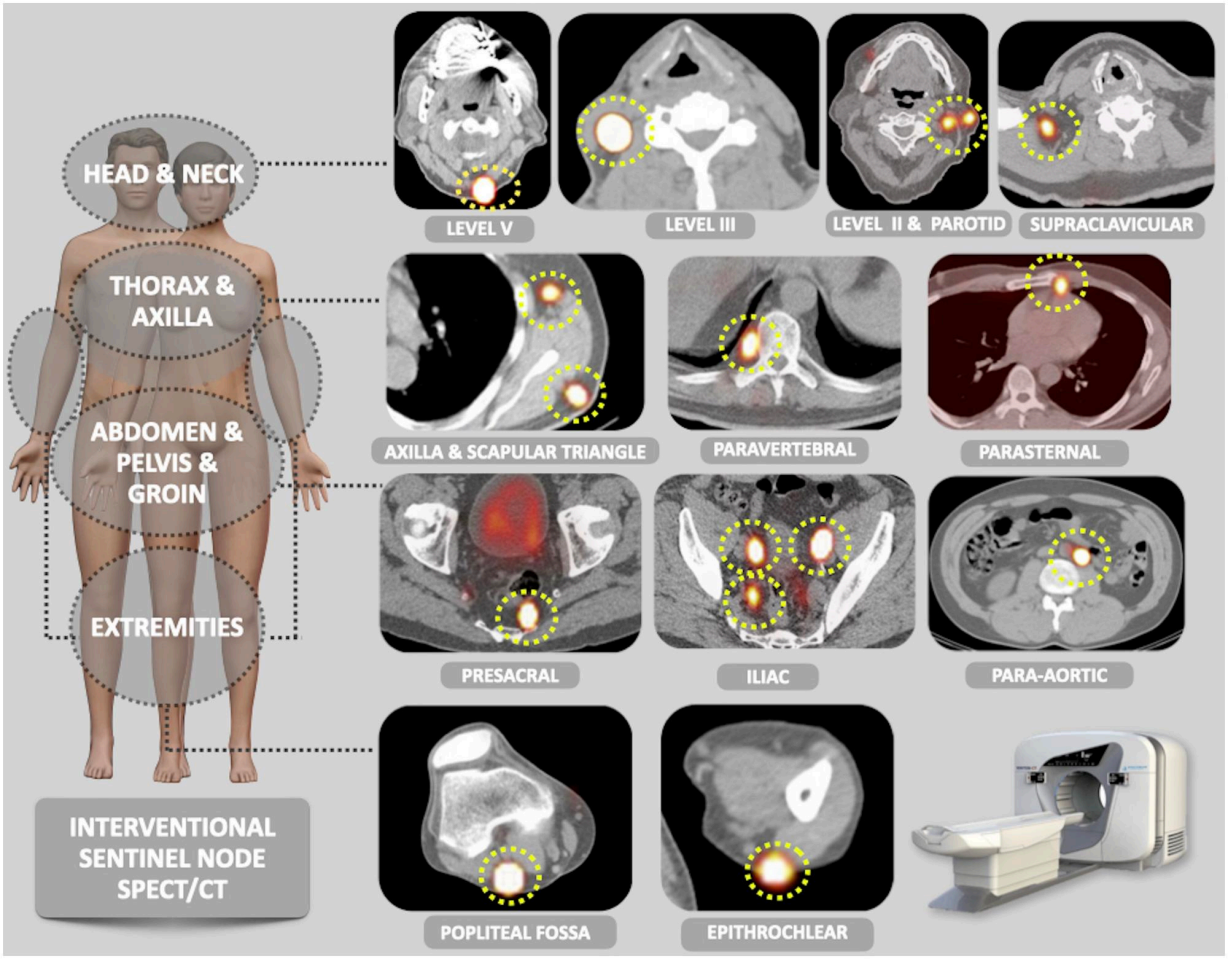
Interventional SPECT/CT overview showing examples of anatomical sentinel node localization in diverse parts of the body (circles).

The development of innovative gamma cameras has facilitated the incorporation of SPECT/CT to preoperative imaging reinforcing the role of iMN in the SN procedure (Figure 3). SPECT/CT is acquired in addition to lymphoscintigraphy in a single preoperative session without additional radiopharmaceutical injection. Although SPECT/CT and lymphoscintigraphy are complementary modalities, SPECT/CT provides specific anatomical landmarks (ie, blood vessels, muscles, lymph node basins, bone structures etc.) to localize SNs already visualized on planar images. Moreover, due to its increased sensitivity SPECT/CT frequently detects additional SNs leading to surgical adjustment. In a prospective multicenter trial coordinated by the International Atomic Energy Agency (IAEA) including 262 melanoma patients, SPECT/CT demonstrated to have clinical value by revealing 70 additional SNs in 53 patients with 25% additional nodes in head and neck melanoma, 26% in upper limb melanoma, 21% in trunk melanoma, and 13% in lower limb melanoma. SPECT/CT did modify the surgical approach with the gamma probe in 97 patients (37%).^14^ Similarly, an adjustment rate (37.4%) for SN surgery guided by SPECT/CT and the gamma probe was found in a recent meta-analysis concerning 17 studies and 1438 melanoma patients.^15^ In a study including 380 patients with melanoma of the extremities, routine SPECT/CT was associated with higher incidence of SN biopsy in lymph node stations (ie, epitrochlear, supraclavicular, infraclavicular, cervical, obturator, popliteal, and external iliac) outside the typical drainage routes from the primary lesions.^16^ In this study, the intraoperative gamma probe adjustment following SPECT/CT in 1181 breast cancer patients was lower than in melanoma (17%) but the number of additional detected SNs increased in 14% when compared with planar lymphoscintigraphy. In another recent study concerning 408 patients with breast cancer, SN identification rate scored almost 99% with 97% solely located in axillary level I; among others, absence of SN detection on SPECT/CT as well as location outside level I and increased SN number were significantly correlated with axillary lymph node metastases.^17^ SPECT/CT is mainly indicated for preoperative SN imaging in patients with ipsilateral cancer relapse, after treatment with breast surgery or radiotherapy, showing a significant higher SN visualization rate and a 60% territory adjustment when compared with planar images of lymphoscintigraphy.^18^ Furthermore, thanks to lymphoscintigraphy and SPECT/CT, roadmaps are generated for SN surgery using intraoperative gamma detection portable devices enabling an accurate resection of SNs localized in the internal mammary chain.^19^^,^^20^

When SPECT/CT is not available, determinate intraoperative devices such as gantry-free freehand SPECT (fhSPECT) and PGC can be used to define preoperative roadmaps over lymphoscintigraphy in melanoma and breast cancer as well as in head and neck cancer.^21–23^

### Head and neck cancers

The lymphatic system of the head and neck includes approximately 250-300 lymph nodes divided into various nodal groups with marked variations in lymphatic drainage depending on the primary site of melanoma or head and neck cancer. For instance, scalp melanomas of the frontal zone drain to different lymph nodes when compared with melanomas of the parietal or occipital areas. Likewise, face and forehead melanomas drain to different basins.^24^ In the oral cavity, malignancies of the lingual apex drain to other groups in comparison with well-lateralized lesions in the tongue or floor of the mouth. Due to this variability in drainage patterns, SPECT/CT in addition to lymphoscintigraphy is crucial not only to accurately identify SNs in an anatomical landscape, but also to detect additional SNs in the vicinity of the primary lesions or in patients with aberrant drainage to different lymph node basins. The procedure is recognized as a standard application for SN surgery. Moreover, SPECT/CT is helpful for the localization of SNs in relation to the surgical neck dissections levels.^25^ In addition, SPECT/CT helps to identify cervical SNs located in levels I and II in patients with primary cancer in floor of mouth or tongue overcoming the shine-through phenomenon. This facilitates subsequent detection at these neck levels with a gamma probe and/or gamma camera in the operating room.^26^ In a study including 66 patients with early-stage cN0 oral cancer, SPECT/CT identified a 22% additional SNs in the neck, and in 20% of the patients with at least one positive SN, the only positive node was detected by SPECT/CT.^27^ At the site of the first echelon nodes indicated by SPECT/CT, the first draining lymph nodes, usually the hottest nodes, indicated by SPECT/CT are harvested and the so-called 10% rule (resecting all lymph nodes with at least 10% of the counts of the hottest excised node) has been found to be useful in the decision-making and to correctly stage the neck.^28^ However, identifying SNs with hand-held gamma probes near the injection site is challenging because of the overshining effect caused by the high activity at the primary lesion. To overcome this limitation, several strategies were adopted, including the use of hybrid tracer such as ICG-^99m^Tc-nanocolloid, ^99m^Tc-tilmanocept, and PGCs which can also help verify the completeness of the SN removal close to the primary tumor.^6^

Also, in head and neck melanoma, SPECT/CT was found to be useful with visualization of 29% additional SNs in the parotid/preauricular region and 44% in level Vb not seen on planar imaging acquired during lymphoscintigraphy.^29^

### Urological malignancies

The 2 most validated urological SN applications are penile and prostate cancer. In penile cancer, SN biopsy has been incorporated in the clinical routine for patients with high-risk (≥pT1b) tumors and cN0 groins are eligible for SN biopsy. In these patients, lymphatic drainage after radiopharmaceutical injection in the glans is mainly bilateral with the inguinal lymph nodes as the first station. Based on RGS dynamic SN biopsy, following preoperative inguinal ultrasound and fine needle aspiration cytology, is associated with a high diagnostic accuracy and low complication rates.^30^ In prostate cancer, SN biopsy could be performed in intermediate- and high-risk patients. Most common pathways of lymphatic distribution after intraprostatic radiotracer injection include the extern iliac and obturator nodes, as well as the internal iliac nodes with less frequent direct pathways to the common iliac, mesorectal and presacral lymph node regions. All lymph node metastases between the level of the iliac bifurcation and the level where the inferior epigastric artery arises from the external iliac artery are considered as regional involvement.^31^ SN biopsy, with mostly laparoscopic RGS as an important component, appears to be comparable to extended pelvic lymph node dissection with a high diagnostic accuracy and a low false negative rate. Recently, SN biopsy using SPECT/CT and laparoscopic gamma probe detection yielded 14% tumor-positive lymph nodes with a 3 mm median metastasis size in 154 patients with a negative PSMA-based PET/CT.^32^ For other urological malignancies like bladder cancer, renal cell carcinoma, and testicular cancer, SN biopsy was performed mostly using laparoscopic RGS but despite acceptable detection rates and sensitivity for identifying lymph node metastases their use remains investigational.^33^

### Gynecological malignancies

Vulvar and cervical malignancies are the most important clinically recognized SN applications.^34^ In vulvar cancer with indication for SN biopsy (unifocal tumors <4 cm, cN0 groins), lymphatic drainage is unilateral or bilateral depending on the primary tumor site and directed to the inguinal nodes. Instead, the lymphatic spread in early-stage cervical cancer may be bilateral because of the midline position of the uterine cervix and the dissemination principally occurs to pelvic stations (ie, parametrial, intern iliac, external iliac, and presacral). For vulvar cancer, a pooled detection rate of 96% was found for lymphoscintigraphy and RGS in a recent meta-analysis including 30 studies.^35^ For cervical cancer, the combination of SPECT/CT and RGS has been found to be highly effective to identify SN metastases with equivalent detection rate in comparison to ICG (92%) but without the possible SN over-identification which may occur with solely fluorescence.^36^

Due to a large methodological variability principally based on the injection, the SN procedure in endometrial cancer has gained popularity only in recent years with gradual incorporation in the clinical routine. Indication is mostly reserved for patients with low-risk/intermediate-risk endometrial cancer with the pelvic pathway as the most common drainage route for tumors located in the middle and lower parts of the uterus, whereas for cancer in the upper corpus and fundus the routes to junctional lymph nodes as well as common iliac and para-aortic nodes are also important. The combination of radiopharmaceutical and blue dye has been found to be highly sensitive^34^ with a high concordance between SPECT/CT and gamma probe findings.^37^ A methodological evaluation showed improved detection rate for planar scintigraphy at 18 hours, higher than SPECT/CT at 1 hour (94% vs. 87%) in 125 patients.^38^

For ovarian cancer SN biopsy remains investigational and the pattern of spread may be multidirectional with possible peritoneal, lymphatic, and hematogenous dissemination. Regarding the lymphatics, para-aortic and the lateral pelvic pathways are the principal routes.^31^ In initial trials, the use of ^99m^Tc-nanocolloid was associated with high detection rates following injection in the ovarian ligaments. The same radiopharmaceutical was also effective in combination with ICG for pelvic and para-aortic SNs.^34^ The combination of radioguidance and fluorescence achieved a detection rate of more than 93%; in this recent study including 63 patients, bimodal detection was performed using a PGC, in addition to the handheld gamma probe, in combination with a NIR-camera.^39^

## Interventional nuclear medicine in the operating room beyond the sentinel node

### Radioguided resection of primary tumors: from ROLL to RSL

The Radioguided Occult Lesion Localization (ROLL) procedure was developed as an alternative to harpoon-guided localization, but with the advantage of being less invasive and based on the use of gamma handheld probes already standardized for the SN procedure in the operating room. In comparative studies, the ROLL procedure was associated with better results than the harpoon technique for resection margins and operating time, but without significant differences for re-excision rates.^40^ Technically, ROLL allows for better centering of the tumor lesion within the specimen compared to other procedures like carbon tracer or harpoon. This is achieved because the intralesional injection of albumin macroaggregates (MAA) labeled with ^99m^Tc, does not migrate from the tumor site after being injected under ultrasound or stereotactic mammography guidance.

The ROLL procedure may be combined with SN biopsy in the same surgical session by means of the injection of a radiocolloid with enough retention in the primary lesion but at the same time presenting some migration to the regional lymph nodes. The procedure is called Sentinel Node and Occult Lesion Localization (SNOLL) and is a safe and precise technique in non-palpable breast cancers.^41^

In recent years, Radioguided Seed Localization (RSL) is becoming an alternative for radioguided excision of primary non-palpable breast tumors, including those patients receiving neoadjuvant systemic therapy (NST). RSL showed comparable results to ROLL in terms of reoperation rates and local recurrence-free survival at 5 years together with a comparable sample weight. In patients with NST, one of the greatest advantages of RSL is that can be performed before NST owing to the long half-life of the implanted iodine-125 (^125^I) seeds. Another potential advantage of RSL, in comparison to ROLL or SNOLL, is the possibility to use more than one seed when needed (eg, tumor bracketing). The ^125^I seeds are inserted under ultrasound or mammographic guidance into the center of the tumor using an 18-G needle and their position is verified by mammography.^42^^,^^43^

The ROLL, and eventually RSL techniques, are also used for the radioguided resection of other solitary lesions such as pulmonary and thyroid nodules.^44^

### Radioguided axillary surgery in breast cancer after neoadjuvant systemic treatment

The introduction of NST in the management of early-stage resectable breast cancer has led to the increasing application of surgical de-escalation regimens in patients with infiltrated lymph nodes. In this context, Targeted Lymph Node Biopsy (TLNB) and Targeted Axillary Dissection (TAD) are playing a significant role for the management of the axilla after NST. Both procedures improve the reduced accuracy of SN biopsy when this is performed as the only modality after NST. For TLNB, the radioactively marked metastatic lymph nodes are selectively removed by RGS after NST is completed. For TAD, the TLNB is combined with SN biopsy.^45^ To mark metastatic lymph nodes in the axilla, the Marking the Axilla with Radioactive Iodine seeds (MARI) procedure is the most widespread in RGS. For MARI, the largest axillary node is selected to insert a ^125^I seed under ultrasound guidance. For surgical excision of the MARI node, the same standard SN equipment is used and after lymph node removal the axilla is checked with a gamma probe or a PGC. The MARI procedure can achieve an identification rate of 97% with a false negative rate of 7%, which is acceptable for predicting the complete response in the affected axilla. When the MARI procedure is combined with SN biopsy in the context of TAD, the identification rate rises slightly to just over 98% but is significantly lower (2%-4%) in terms of false negative rate.^46^ Apart from its high efficacy and reliability in implementing TLNB and TAD, the MARI procedure is associated with a minimal risk of ^125^I-seed displacement in the time between insertion and surgery. On the other hand, the MARI procedure in combination with PET/CT using ^18^F-labeled fluorodeoxyglucose (^18^F-FDG) is used to stratify the risk in the axilla to reduce subsequent total lymph node dissection enabling its replacement by a personalized treatment.^47^ This approach of response-guided axillary treatment using the MARI-protocol resulted in a 5-year very low recurrence risk in patients with limited axillary nodal disease (≤3 positive axillary nodes on ^18^F-FDG PET/CT).^48^ Likewise, the oncologic outcome was excellent in patients with extensive nodal involvement (≥4 ^18^F-FDG positive axillary nodes) and omission of full lymph node dissection who achieve complete response of the MARI node after NST.^49^

### Radioguided resection of oligometastases

In patients with isolated distant metastases, iNM is useful for both diagnosis and subsequent resection by means of RGS.^50^ This is because nuclear medicine provides various diagnostic whole-body image modalities capable of tracking metastases presenting radiopharmaceutical uptake. In the era prior to the introduction of PET, other conventional nuclear medicine modalities were very helpful in achieving this goal, for example in the case of solitary lesions in the skeleton visualized on bone scintigraphy and selected for RGS in patients with high clinical suspicion of recurrence.^51^

**Figure 4:**
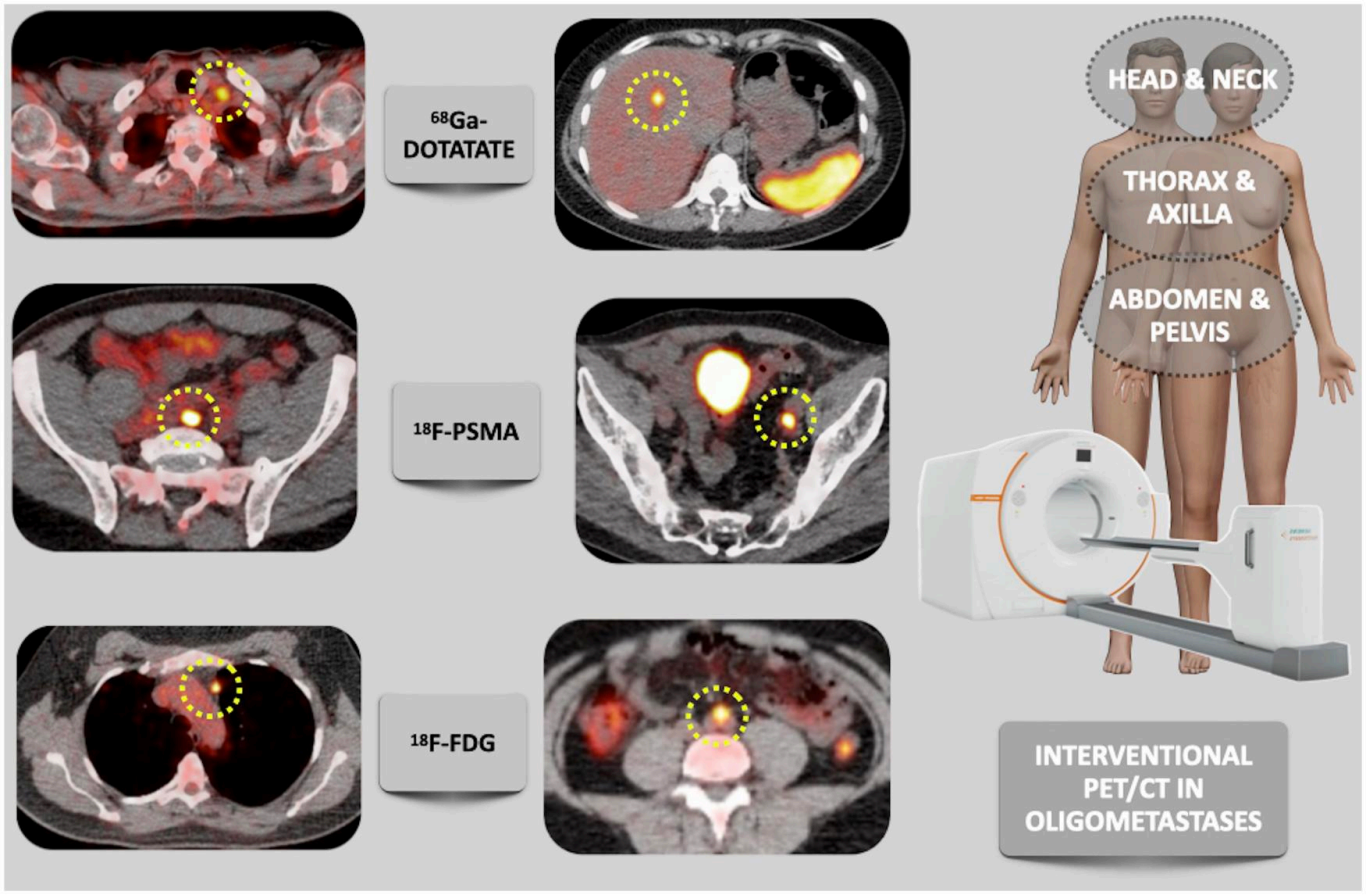
Examples of isolated metastases localized with tumor-seeking PET-tracers (circles) illustrating the potential of preoperative Interventional PET/CT imaging to generate precision roadmaps for their radioguided resection.

In the last 2 decades, the incorporation of various tumor-seeking PET tracers has reinforced the diagnosis of distant metastases in cancer patients (Figure 4). For instance, ^18^F-FDG, widely used in breast cancer and other malignancies, is becoming the imaging standard in the diagnosis of extension. Recently, the role of PET/CT is reinforced by other PET radiopharmaceuticals with greater accuracy for detecting distant metastases and guiding surgery in oligometastastic disease. Among them, fibroblast activating protein inhibitors (FAPI) ligands labeled with ^68^Ga or ^18^F, ^18^F-FES (fluoroestradiol), prostate-specific membrane antigen (PSMA) ligands labeled with ^68^Ga or ^18^F, DOTA-peptides labeled with ^68^Ga and HER2-targeted radiopharmaceuticals such as ^89^Zr-trastuzumab.^52^^,^^53^

Although PET/CT imaging is used when clinical recurrence is suspected for patient selection by localizing isolated metastases to guide surgery, the direct use of PET-tracers for surgery in the operating room has been limited due to the low detection sensitivity of high-energy gamma devices.^6^ This has led to a 2-tracer approach using RGS with standard equipment in the operating room.^54^ This strategy has been extensively validated in prostate cancer by replacing the PET radionuclide (eg, ^68^Ga, ^18^F) with a SPECT (^99m^Tc or ^111^In) radionuclide, thereby facilitating the use of low-energy gamma probes or PGSs in the operating room. Using this approach, RGS with ^99m^Tc-PSMA enabled resection 343 out of 364 (94%) prostate cancer recurrence patients with biochemical progression and detected lesions on PSMA-PET/CT.^55^

### Tumor resection and radioguided *ex vivo* margin assessment

RGS can help surgeons remove malignant tumors while ensuring that no cancerous tissue is left at the margins. In addition to radioguided tumor resection, control of specimen borders is becoming an emerging area of interest for iNM and RGS. As already described in this review probably most common applications have been based on ROLL and RSL following local administration of a radioactive tracer. For malignancies of breast, lung, thyroid and others, resection control is accomplished using gamma counting of the specimen in comparison to background radioactivity.^44^ More recently a second model of radioguided lesion excision and specimen border control related to systemic radiotracer administration has gained in popularity.^50^ When PET-tracers are used an innovative approach is based on the use of detection probes working with beta radiation which has a lower penetration (a few millimeters) compared to gamma radiation. These beta probes are more apt to discriminate the signals from the tumor margins from the nearby healthy tissues. Among other malignancies, this device was successfully tested in a feasibility study with ^18^F-FDG in cervical cancer.^56^ Also Cerenkov luminescence imaging of ^68^Ga-PSMA was used for margin assessment in prostate cancer^57^ as well as a recently introduced micro-PET/CT device for the evaluation of surgical specimens in the operating room in surgically excised head and neck malignancies in patients receiving ^18^F-FDG.^58^ This novel specimen PET/CT device has also been used to assess radical prostatectomy specimens in high-risk prostate cancer who have received PSMA labeled with ^68^Ga or ^18^F.^59^ From the gamma RGS side, a novel modality has been proposed combining fhSPECT with Light Detection and Ranging. This technique allows to display the radioactive signal within the surface-contours of the excised specimens, and was used to obtain *ex vivo* scans of the prostate cancer tissues exploiting ^99m^Tc-PSMA-I&S signal (Imaging & Surgery).^60^^,^^61^

## Local and systemic radiotracers for nuclear medicine imaging and radioguided surgery

With the development of the SN procedure for clinical applications more than 30 years ago, radiocolloids were incorporated to depict lymphatic drainage and SN identification following their administration around the primary tumor. At that time, existing registered radiocolloids were preferred leading to significant intercontinental variability because of differences in particle size. These first generation radiocolloids remains the most widely used for surgical SN biopsy. In Europe, several albumin nanoparticle-based radiopharmaceuticals are available, ranging from Nanocoll (Sorin/GE Healthcare), which contains at least 95% of particles measuring less than 80 nm in diameter, to SentiScint (Medi-Radiopharma), which has more than 80% particles measuring 100-600 nm. Other products with particle distribution approximately similar to Nanocoll include Nanotop (Rotop), Nanoscan (Medi-Radiopharma), and Nanoalbumin (Medi-Radiopharma). In recent years, the fluorophore ICG has been incorporated to Nanocoll, Nanotop, and Nanoscan to generate hybrid tracers suitable for bimodal SN detection in both open and (robot-assisted) laparoscopic surgery.^62^^,^^63^^99m^Tc-tilmanocept was more recently introduced. It binds to mannose receptors expressed on the surface of lymph node macrophages and dendritic cells reducing its migration to higher echelon nodes, despite a 7 nm diameter particle size. Its use for SN surgery is increasing in last years.^64^

Concerning intravenously administered systemic tumor-seeking radiopharmaceuticals—such as ^99m^Tc-sestamibi for SPECT/CT-guided resection of parathyroid adenomas, iodine-123 for metastases in thyroid cancer recurrence, and ^99m^Tc-biphosphonates for image guided resection of bone lesions- recent RGS applications have increasingly relied on PET/CT radiopharmaceuticals, including ^18^F-FDG, PSMA-ligands, DOTA-peptides, and more recently FAPI-ligands.^7^ These radiopharmaceuticals allow detection of cancer recurrence when indicated by biochemical markers, and guide subsequent radio-tagged resection of identified malignant lesions. In this context, the original β^+^ radionuclide may be replaced by ^99m^Tc or ^111^In to enable SPECT/CT radioguided resection. This RGS approach was successful as above mentioned^51^ but SPECT/CT did not detect all PSMA-expressing lesions depicted by PET/CT. In this setting, efforts have been made to incorporate fluorophores to PET tracers to facilitate intraoperative bimodal detection. All these possibilities have recently been reviewed together with the potential use of beta radiation probes which can work with the same PET/CT tracer used in the initial diagnosis.^65–67^

## Technological equipment and advances for new surgical challenges

### Detection probes and portable gamma cameras

Although the concept of RGS originated several decades ago, it was only with the introduction of the SN procedure that its use increased with the handheld gamma probe as an central tool in the operating room.^1^ Basic considerations and criteria for the use of intraoperative gamma detection devices are based on some essential parameters such as sensitivity (or efficiency), energy resolution, contrast, and spatial resolution.^68^ These parameters have been recently been discussed in relation to new devices for RGS classifying detection probes in 4 categories: standard gamma probes working with low- to mid-energy (≤400 keV), high-energy gamma probes suitable for >400 keV, β^+^ probes for detection of positrons, and β^−^ probes for detection of electrons.^6^

Currently, the standard gamma probe is used for more than 90% of RGS procedures based on ^99m^Tc and to a lesser extent ^125^I, ^123^I, and ^111^In. Other detection probes are principally used for investigational objectives. However, despite the efficiency of the standard gamma probe, its main limitation is the inability to precisely localize the signal. This limits the detection of small lesions, principally those located near the radiotracer injection.

The limitations of gamma probes have led to the development of PGCs which enable to reduce the localization time of targeted tissues by allowing 2D mapping in a larger field of view. PGC-generated images from different directions increase the diagnostic accuracy of overlapping lesions or identifying lesions close to the injection site. However, PGC maneuverability is limited in the operating room due to its more voluminous configuration compared to gamma probes. PGCs are also designed for working with low- to mid-energy gamma ray detection and are becoming widespread due to their high-resolution SN imaging. The development and outlook of intraoperative PGC is in progress not only as a single modality but also in a bimodal configuration.^69^ The newest PGC models are based on CZT (cadmium-zinc-telluride) technology which has resulted in improved sensitivity for gamma-ray detection.^6^

### Intraoperative innovative devices: toward robot-assisted procedures

Concerning precision RGS, most tool innovations have been oriented to optimize existing approaches. An example concerns the development of fhSPECT which can generate 3D scanning of the radiopharmaceutical distribution in the targets within the patient. By means of a surgical navigation system, the position and orientation of the gamma detector can be followed during gamma tracing of the targeted tissue in the operating room. Originally introduced using a standard gamma probe this technology was extended to handheld gamma cameras and other intraoperative detection devices.^70^ This modality was evaluated for different RGS procedures concerning SN surgery in breast cancer, head and neck cancers, melanoma, urological and gynecological malignancies as well as RSL in breast cancer, ROLL for pulmonary lesions, PSMA-targeted prostate lesions, parathyroid adenoma, neuroendocrine tumors and bone lesions.^71^

Advances in gamma detection concerns not only devices for open surgery. Recently, for robot-assisted laparoscopy in the context of RGS an adjustment of nuclear medicine contribution has been effectuated with the incorporation of flexible miniaturized gamma probes (Drop-In devices) replacing the rigid laparoscopic gamma probe model introduced at the end of the past century. Drop-In devices, currently commercially available for minimally invasive surgery, enable a superior detection rate and greater angle-maneuverability for pelvic SN detection.^72^^,^^73^ A further incremental development inspired by the Drop-In concept is the recently designed Click-On probe which allows integration of the device in the same pincers of the robot. Due to a better dexterity, the Click-On device appears to provide a 40% reduction in movements compared with the Drop-In probe.^74^

## Multiplexing modalities: the future is hybrid

Currently, iNM and RGS are the standard in guiding many precision surgery procedures. To overcome the limitations of gamma and beta counting in complex anatomic areas, the multiplexing concept was introduced as a future modality based on the complementary information that other signatures provide to the iNM procedure. This concept is called hybrid, bimodal or multimodal procedures adding optical and NIR-detection modalities to RGS in the operating room.^75^ Following the development of hybrid tracers combining fluorescence with radioactivity, one of the requirements for bimodal detectability is the availability of adequate NIR-equipment associated to RGS practice. In many centers, NIR-cameras are used by surgeons as an independent modality. For open SN surgery in axilla and groin, a standard gamma probe in combination with a simple NIR-camera allowing acoustic gamma signal together with high resolution fluorescence imaging may be enough. In sites of complex lymphatic drainage (ie, head and neck, certain regions of the trunk), a high-resolution PGC added to the above-mentioned equipment, and if available, a second generation NIR-camera allowing real time work with room light obtaining color fluorescent images are recommended.^53^

For laparoscopic SN surgery, the most commonly used combination is a gamma probe with long stylus and a NIR-camera, both rigid models of standard laparoscopic use. Their use during the procedure is often alternated, with the gamma probe being actuated separately by the nuclear medicine physician. By the integration of the fluorescence signal to the console, its visualization was simplified giving functional space to the rigid laparoscopic gamma probe and recently to the flexible Drop-In probe.^72^^,^^73^ Hybrid surgery for resection of metastasis or tumor recurrence, administering systemic radiotracers can be performed with the same equipment used for hybrid SN surgery. The tendency to incorporate fluorescence to systemic radiotracers will extend rescue surgery in patients with oligometastatic progression.^4^ There is continuous improvement in the development of hybrid tracers for surgical guidance. For instance, the recently developed ^99m^Tc-PSMA-HSG (Hybrid SPECT/Fluorescence or Hybrid Surgical Guidance) showed a 50% increase in tumor uptake compared with the previously used ^99m^Tc-PSMA-I&S.^61^ Another future development is the integration of hybrid detection systems with a surgical navigation device capable of producing 3D fluorescence tomography reconstructions in addition to fhSPECT modality.^76^ In this approach, the surgical targets are defined based on the 3D preoperative SPECT/CT or intraoperative fhSPECT images. The tracked fluorescence camera is navigated toward these targets, using virtual or augmented reality displays of the SPECT findings in the fluorescence video display. The potential inaccuracies of the navigation workflow are corrected via the real time fluorescence feedback.

The multiplexing concept is emerging and might become an important fundament for new tools and approaches concerning iNM and RGS in the future as recently illustrated by the use of fhSPECT in combination with handheld Light Detection and Ranging as a novel hybrid modality to display radioactive volumes within the surface-contours of the excised specimens.^77^

## Conclusion

Both iNM and RGS today play an important role in precision surgery for different malignancies such as breast cancer, melanoma, head/neck cancer as well as urological and gynecological malignancies. Most applications are related to radioguided SN procedures which have been incorporated in current protocols endorsed by various scientific associations enabling a significant reduction in the extent of surgery. Besides SN surgery both the excision of primary tumors and the resection of isolated distant metastases can currently be performed using iNM and RGS.

Following this clinical extension of iNM and RGS in precision surgery as discussed in this review, methodological adjustments in protocols have facilitated a gradual incorporation of several technological advances such as the development of more specific radiopharmaceuticals and hybrid detection concepts that combine radioguidance with eg, fluorescence imaging. Likewise, in a future perspective the integration of image guidance in minimally invasive (robotic and non-robotic) procedures as well as the use of surgical navigation approaches including augmented and virtual reality displays will reinforce the role of nuclear medicine in the operating room.
